# Evaluation of In Vitro Activity of Essential Oils against *Trypanosoma brucei brucei* and *Trypanosoma evansi*

**DOI:** 10.1155/2010/534601

**Published:** 2010-03-28

**Authors:** Nathan Habila, Abel S. Agbaji, Zakari Ladan, Isaac A. Bello, Emmanuel Haruna, Monday A. Dakare, Taofiq O. Atolagbe

**Affiliations:** ^1^Biochemistry Division, National Research Institute for Chemical Technology, P.M.B 1052 Zaria, Nigeria; ^2^Department of Chemistry, Ahmadu Bello University, P.M.B 1045 Zaria, Nigeria; ^3^Department of Biochemistry, Ahmadu Bello University, P.M.B. 1045 Zaria, Nigeria

## Abstract

Essential oils (EOs) from *Cymbopogon citratus* (CC), *Eucalyptus citriodora* (EC), *Eucalyptus camaldulensis* (ED), and *Citrus sinensis* (CS) were obtained by hydrodistillation process. The EOs were evaluated in vitro for activity against *Trypanosoma brucei brucei* (Tbb) and *Trypanosoma evansi* (*T. evansi*). The EOs were found to possess antitrypanosomal activity in vitro in a dose-dependent pattern in a short period of time. The drop in number of parasite over time was achieved doses of 0.4 g/ml, 0.2 g/mL, and 0.1 g/mL for all the EOs. The concentration of 0.4 g/mL CC was more potent at 3 minutes and 2 minutes for Tbb and *T. evansi*, respectively. The GC-MS analysis of the EOs revealed presence of Cyclobutane (96.09%) in CS, 6-octenal (77.11%) in EC, Eucalyptol (75%) in ED, and Citral (38.32%) in CC among several other organic compounds. The results are discussed in relation to trypanosome chemotherapy.

## 1. Introduction

African Trypanosomosis is a disease caused by several trypanosomes of the Trypanosomatidae family, which affect humans or animals. *Trypanosoma evansi* (*T. evansi*) is the most widely distributed of the pathogenic African trypanosomes, affecting domestic livestock and wildlife [1] and causes African trypanosomosis, a disease which affects humans and animals [2–4]. It is also well known that *T. evansi* is the etiological agent of a disease called Surra which is a major cause of livestock morbidity and mortality [5, 6] and it has been reported to occur in a variety of hosts the most economically important being horses, buffalo and cattle [3, 6, 7]. *Trypanosoma brucei brucei* (Tbb) also causes African Trypanosomosis along with several other species of trypanosomes although Tbb has morphological similarities with *T. evansi*, the latter differs from related species by the absence of kinetoblast DNA minicircle [8]. 

Currently, 35 million people and 25 million cattle in Africa are at risk of contracting the disease which is fatal if untreated [9]. Ironically, some registered trypanocides are frequently toxic, require lengthy administration, lack efficacy and are sometimes unaffordable for most of the patients [10].

In many African countries, plants have traditionally been used for centuries and are still being widely used to treat this illness and other parasitic diseases which may be due to limited availability and affordability of pharmaceutical products. Essential oils (EOs) are odoriferous constituents produced by plants and have been traditionally used for respiratory tract infections and are known to possess antimicrobial properties [11, 12]. It is also well known that some EOs have some chemical components with insecticidal properties [13] and some EOs have characteristic array of compounds in different variations of which the most abundant types of chemical compounds present are Terpenoids and Phenyl compounds [14]. 

The present study, investigates the evidence of in vitro antitrypanosomal activity of EOs from *Cymbopogon citratus, Eucalyptus citriodora, Eucalyptus camaldulensis* and *Citrus sinensis* against Tbb and *T. evansi* in an attempt to explore natural products that may be more accessible sources of trypanocides Vis-a-vis species difference and the fact that current available treatments are far from being ideal.

## 2. Materials and Methods

### 2.1. Collection of Plant Materials

Fresh *Citrus sinensis* (CS) peels were obtained from fresh oranges purchased in Zaria-Nigeria in April 2009. The Leaves of *Cymbopogon citratus *(CC) were harvested in the Research Garden of National Research Institute for Chemical Technology (NARICT) Zaria-Nigeria in March 2009*, Eucalyptus Camaldulensis* (ED) and *Eucalyptus citriodora *(EC) were harvested from state forestry Shika, Kaduna state—Nigeria in April 2009. The plants (CC, EC, and ED) were identified at department of Biological sciences, Ahmadu Bello University, Zaria-Nigeria.

### 2.2. Test Organisms

The parasites *Trypanosoma brucei brucei* and *Trypanosoma evansi* were used and obtained from stabilates maintained at the parasitology laboratory, Faculty of Veterinary medicine, Ahmadu Bello University, Zaria-Nigeria.

### 2.3. Extraction of Essential Oils

Briefly, 250 g fresh plant material of each plant was put in a round bottom flask and 1000 mL distilled water was added before subjecting to hydro distillation [15] for 6 hours. The EOs were recovered and dried over anhydrous sodium sulphate. Each EOs produced was diluted using pure vegetable oil obtained from Grand cereals and oil mills limited, United African Company, Lagos-Nigeria. The different concentrations of EOs were prepared at 0.4 g/mL, 0.2 g/mL and 0.1 g/mL in the diluent constituted into 50%, 25%, and 1%, respectively.

### 2.4. Determination of Parasiteamia

The parasites were maintained in the laboratory by continuous passage in rats until it is required. The passage was done when parasiteamia was in the range of 16–32 parasites per field with infected blood containing 1 × 10^3^ parasites was introduced intraperitoneally into healthy rats in 0.1–0.2 mL blood/physiological saline solution. The numbers of parasites were determined microscopically at magnification ×400 and Parasitaemia was monitored daily.

### 2.5. In Vitro Test

The Drug Incubation Infectivity Test [16] with Wet and thick blood films method [17] to detect any motile trypanosomes was done in triplicates in a 96 well microtitre plate with 50 *μ*L of infected blood was incubated with 20 *μ*L of each EOs from 50%, 25% and 1% (previously prepared) giving a final concentration of 114 mg/mL, 57 mg/mL and 28.6 mg/mL, respectively. Parasitaemia was monitored between 1–20 minutes of incubation at 30°C. About 0.5 *μ*L–1 *μ*L of test mixtures were observed every 1 minute under a microscope ×400. A set of Positive Control (PC), Negative Control (NC) and Diluent Control (DC) were set up to stand for 4hrs with the infected blood containing the parasites. The PC contained 25 mg/mL of DIMINAVETO^R^ (1.05 g of Diminazene diaceturate and 1.31 g Antipyrine), the NC contained only the infected blood suspended in heparin and Phosphate Buffer Saline Glucose (pH 7.2) and the DC contained only the pure vegetable oil (100%).

### 2.6. Gas Chromatography-Mass Spectroscopy (GC-MS)

This was carried out as described by Maciel et al. [13] using EOs obtained from CS, CC, EC, and ED in GC-MS QP2010 SHIMADZU, Japan. The Column oven temperature was 50°C, Injection temperature was 200°C, Pressure was 53.2 kPa., Linear velocity was 36.2 cm/sec and Total flow was 53.8 mL/min. The Compounds were identified by GC retention time (RT) and by comparism with those present in the National Institute for Standard Technology computer data bank (NIST; 05 s. LIB).

## 3. Results and Discussion

**Figure 1:**
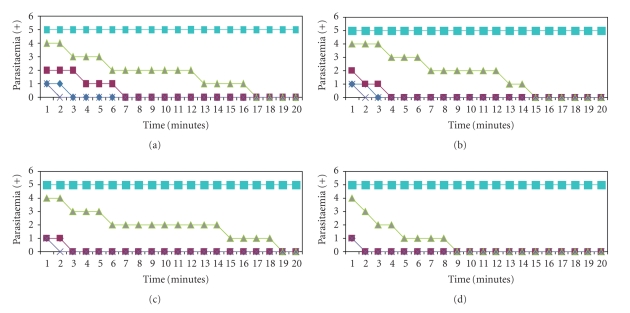
Effect of CS (a), ED (b), EC, (c) and CC (d) essential oils (EO) on *T. evansi* blood forms. The EOs were diluted at 0.4 g/mL (dark blue), 0.2 g/mL (red) and 0.1 g/mL (green) in pure vegetable oil as diluent and then added to the cultures, as described previously. Treatment of the parasites with Diminaveto (Positive control, purple) induced total lysis after 2 minutes. The number of parasites remained constant in negative controls and diluent control (light blue).

**Figure 2:**
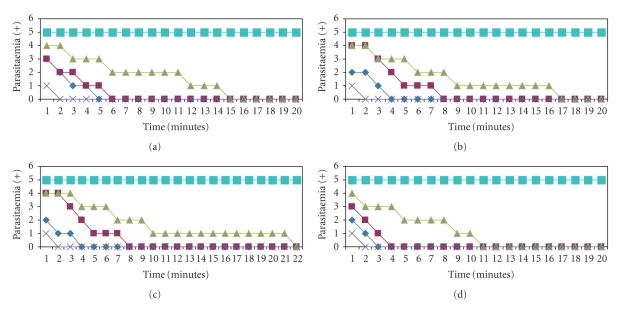
Effect of CS (a), ED (b), EC (c) and CC (d) essential oils (EO) on Tbb blood forms. The EOs were diluted at 0.4 g/mL (dark blue), 0.2 g/mL (red) or 0.1 g/mL (green) in pure vegetable oil as diluent and then added to the cultures, as described previously. Treatment of the parasites with Diminaveto (Positive control, purple) induced total lysis after 2 minutes. The number of parasites remained constant in negative controls and diluent control (light blue).

The results in Figures 1 and 2 show the in vitro activity of EC, CS, ED and CC on *T. evansi* and Tbb. In the results presented in Figures 1(a), 1(b), 1(c) and 1(d), it was observed that the dose of 0.4 g/mL concentration of each of the EOs used caused death of the parasite in 3 minutes (except for CC which was in 2 minutes) against *T. evansi*. When the concentrations of the EOs were 0.2 g/mL and 0.1 g/mL, the time of parasite death for CS was 7 minutes and 17 minutes, ED was 4 minutes and 15 minutes, EC was 3 minutes and 19 minutes, and CC was 2 minutes and 10 minutes, respectively. Figures 2(a), 2(b), 2(c), and 2(d) states the in vitro Antitrypanosomal activity of the four species of EOs against Tbb where 0.4 g/mL concentration induced death of Tbb in 5 minutes, 4 minutes, and 3 minutes for CS, ED/EC and CC. When the concentrations of the EOs were 0.2 g/mL and 0.1 g/mL, the time of parasite death for CS was 6 minutes and 15 minutes, ED was 8 minutes and 17 minutes, EC was 8 minutes and 22 minutes, and CC was 4 minutes and 11 minutes, respectively. This indicates a dose and time-dependent pattern of parasite death in both *T. evansi* and Tbb. The result shows that CC had a higher effect in decreasing cell number more than ED, EC, and CS against both *T. evansi* and Tbb. All EOs showed activity on both parasites even at 0.1 g/mL concentration but there was variation in the decreased in cell number of the candidates EO. 

Table 1 shows the various compounds present in the EOs and the chemical compositions were found to be different for each EO. For example, Citral (38.32%) was present in CC, Beta-myrcene (1.81%) and Cyclobutane (98.08%) was present in CS, Eucalptol (75.04%) was present in ED and 6-Octenal (77.11%) was present in EC. Most of these compounds like, Beta-myrcene, Eucalyptol and Citral are known to have biological activity [13, 18, 19] which are also raw materials against plants and human pathogens [20]. Some reports have also shown that CC play a role in peroxidation by inhibiting free radical attacks on biomembranes [21, 22] especially the red blood cells, and are not likely to pose any health risk [15]. The activity of EOs could also be due to the hydrophobic nature of the cyclic hydrocarbons (Table 1), which allow EOs to interact with the Trypanosomes causing conformational changes in the parasite membrane structure resulting to loss in membrane stability [21]. 

The quick decrease in parasite number in the in vitro tests for both Tbb and *T. evansi *indicates that the EOs is in fact killing the parasites, by an unknown mechanism although EOs can have multiple modes of action and multiple points of disruption [23] like inhibiting some key enzymes in the parasite glycolytic pathway. It is also known that some EOs components inhibit acetylcholinesterase activity and act on other vulnerable sites, such as cytochrome p450 [13]. This multicomponent nature of plant EOs is an advantage for several target sites on trypanosomes, which is especially relevant at a time of increasing emergence of multi-resistant pathogens. 

We did not observe any resumption of flagellate movement or increase in cell number of Tbb and *T. evansi* after 4 hours incubation and observation with the PC, CC, ED, EC and CS. The NC and DC had no effect on the parasites or cell number (Figures 1 and 2) and this gives credence to the impetus of all the EOs species used. The observations from Figures 1 and 2 indicates that species difference of Trypanosomes may be responsible for the efficacy of Trypanocides in line with the fact that all the EOs differ in the way the parasite number decreases with time.

In this study, the evaluation of the four EOs species was able to decrease cell number of Tbb and *T. evansi* in a dose dependent pattern. The EOs from CC had a greater effect of decreasing parasite number more than EC, CS and ED but understanding the real mechanism of action will require further investigation. Sequel to these qualities of EO, it would be expedient to subject it for further trypanosome pathophysiology.

## Figures and Tables

**Table 1:** GC-MS analysis for essential oils from CS, ED, EC and CC.

Compound	RT	CS(%)	ED(%)	EC(%)	CC(%)
Beta-Myrcene	10.045	1.81	—	—	—
Cyclobutane	11.322	96.08	—	—	—
1, 6-octadien-3-ol	13.186	1.70	—	—	—
Decanal	15.355	0.41	—	—	—

6-octenal	14.405	—	—	77.11	—
Cyclohexanol	14.573	—	—	3.42	—
6-octen-1-ol	15.878	—	—	14.09	—
2, 6-Octadiene	17.790	—	—	2.58	—
Cyclohexanol	17.899	—	—	1.11	—
Bicyclo[7.2.0]undec-4-ene	18.968	—	—	1.29	—
1, 7-Nonadiene	25.464	—	—	0.40	—

Bicyclo[3.1.1]hept-2-ene	8.490	—	10.27	—	—
Benzene	11.129	—	6.23	—	—
Cyclohexene	11.217	—	4.92	—	—
Eucalyptol	11.452	—	75.04	—	—
p-meth-1-en-8-ol	15.261		1.65		
2-Oxabicyclo[2.2.2]octan-6-ol	17.610	—	1.09	—	—
(2S, 4R)-P-metha-[1(7), 8]-diene-2-hydroperoxide	21.253	—	0.80	—	—

2, 6-octadien-1-ol	18.717	—	—	—	26.63
2, 6-octadienal	19.242	—	—	—	35.05
Citral	18.408	—	—	—	38.32
